# Rearranging to resist cell death

**DOI:** 10.7554/eLife.104942

**Published:** 2024-12-16

**Authors:** Dominik Brokatzky, Serge Mostowy

**Affiliations:** 1 Department of Infection Biology, London School of Hygiene & Tropical Medicine London United Kingdom

**Keywords:** giant unilocular vacuole, anoikis, macropinocytosis, extracellular matrix detachment, F-actin, Other

## Abstract

Cytoskeleton rearrangements promote formation of a giant structure called a GUVac that stops cells from dying when they become detached from the extracellular matrix.

**Related research article** Kim J, Kim D, Kim DK, Lee SH, Jang W, Lim DS. 2024. Formation of a giant unilocular vacuole via macropinocytosis-like process confers anoikis resistance. *eLife* **13**:RP96178. doi: 10.7554/eLife.96178.

For cells to survive, they must maintain contact with their neighbouring cells or the network of proteins and molecules surrounding them known as the extracellular matrix. The cytoskeleton – which includes a dynamic network of actin filaments and associated proteins – plays an important role in sensing when cells disconnect from the extracellular matrix. If a cell detaches, this triggers a type of programmed cell death known as anoikis, which ensures cells do not reattach at other locations (Frisch and Francis, 1994).

Becoming resistant to anoikis is a key step in tumor metastasis, as it allows cells to spread and invade other tissues (Adeshakin et al., 2021; Kim et al., 2012). However, the mechanisms underlying anoikis resistance are poorly understood. Now, in eLife, Wonyul Jang and Dae-Sik Lim and colleagues – including Jeongsik Kim and Dahyun Kim as joint first authors – report a previously unrecognised cell survival mechanism that induces anoikis resistance (Kim et al., 2024).

The team (who are based at the Korea Advanced Institute of Science and Technology and Seoul National University) used state-of-the-art techniques to study epithelial cells that had been cultured in suspension and therefore were not attached to any extracellular matrix. This was done by exposing them to a solution called trypsin-EDTA, which detaches cells from their surface. They found that treating these cells with the toxin latrunculin B, which inhibits the formation of actin filaments, caused parts of the cell membrane to curve inwards and form structures known as vacuoles. As these vacuoles accumulated in the cytoplasm, they combined to form a giant unilocular vacuole (known as a GUVac), which was between 0.5 and 1 µm wide (Figure 1). The finding suggests that the breakdown of actin filaments, also known as depolymerization, is required for GUVac formation. This differs from how vacuoles form through conventional macropinocytosis, in which actin is polymerized, and the plasma membrane forms outward protrusions (Swanson, 2008).

**Figure 1.**
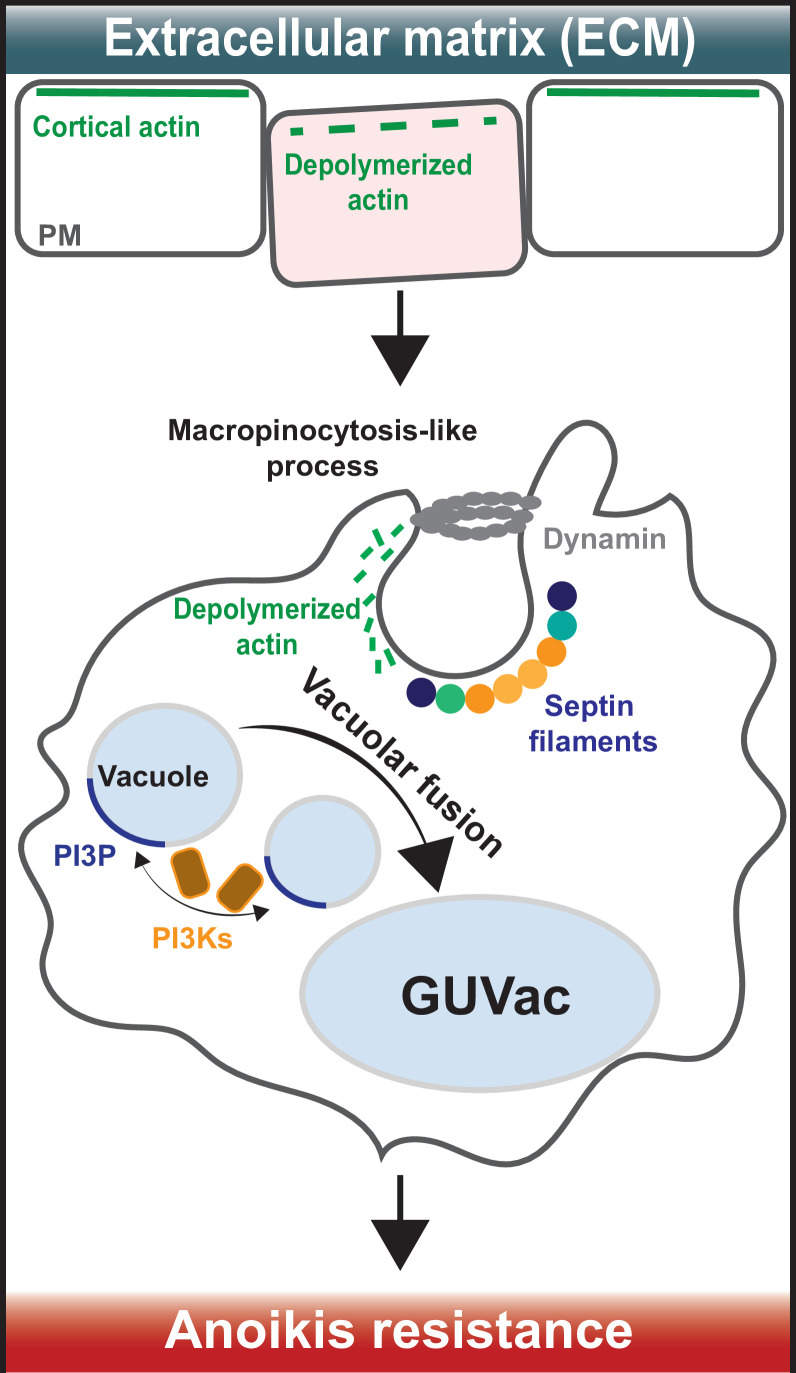
How giant unilocular vacuoles that prevent cell death are formed. (Top) When cells are attached to the extracellular matrix (ECM), their cortical actin (which is linked to the plasma membrane) interacts with proteins in the surrounding ECM to stabilize the plasma membrane (PM). When cells detach from the ECM, cortical actin depolarizes and breaks down, causing micron-scale inward curvatures to form in the plasma membrane. (Bottom) These invaginations promote the recruitment of septin proteins (coloured circles) to the inwardly curved regions of the plasma membrane. At the same time, a protein called dynamin (grey ring) cleaves the invaginations from the membrane to form structures known as vacuoles (small light blue circles) that then accumulate inside the cell. Vacuole formation is also aided by the molecule PI(3)P (dark blue), which is synthesized by enzymes known as PI3Ks (brown squares with orange outline). These processes eventually cause the vacuoles to merge with one another and form a giant unilocular vacuole known as GUVac (large light blue oval) which promotes resistance to anoikis.

Next, Kim et al. set out to find if a group of proteins called septins – which are best known for their roles in cell division and cell-autonomous immunity (Curtis and Gladfelter, 2024; Mostowy and Cossart, 2012) – may be involved in GUVac formation. Septins are an unconventional component of the cytoskeleton which attach to micron-sized curvatures within the plasma membrane and assemble into filaments that bind to actin-based structures. Kim et al. used small interfering RNAs to deplete two septin family members – called SEPT2 and SEPT9 – in suspended cells treated with latrunculin B. This reduced the formation of GUVacs, suggesting that septins have a role in their generation. This finding is interesting considering recent work showed that septin filaments help a human cancer cell line to anchor their actin filaments to the plasma membrane (Martins et al., 2023).

Septins also bind to a family of lipids called phosphoinositides, which are found in eukaryotic membranes and have roles in many cellular processes. Kim et al. found that a phosphoinositide called PI3P – which is produced by enzymes known as VPS34 and PI3K-C2α during vacuole fusion – is crucial for GUVac formation. The role of septins in GUVac formation was further supported by experiments treating the epithelial cells with a drug called forchlorfenuron. Inhibiting septin assembly and function with this drug reduced septin recruitment to the plasma membrane, which suppressed GUVac formation. It will be interesting to see how other, more novel compounds that inhibit or stabilise septin assembly impact the generation of GUVacs, which could offer new insights into the role of septins in this process (Kim et al., 2020; Princen et al., 2024).

Strikingly, using suspended cells treated with latrunculin B, Kim et al. found that epithelial cells with defects in GUVac formation – due to lacking the genes for PI3K-C2α and VPS34 – were highly susceptible to cell death. This highlights a critical role for GUVac formation in anoikis resistance when cells are detached from the extracellular matrix and their neighbouring cells.

The findings of Kim et al. significantly advance our understanding of anoikis resistance by identifying GUVac formation as a novel strategy for cell survival. However, many questions remain. For example, is GUVac formation a conserved functional program across mammalian cells, or is it specific to certain cell types? Kim et al. focused on mammary epithelial cell lines treated with the actin inhibitor latrunculin B, leaving the broader relevance of GUVac formation uncertain. Furthermore, it is still unclear precisely how GUVacs protect cells from anoikis.

The work of Kim et al. also highlights the potential for targeting components of the cytoskeleton as a way to treat cancer. It is therefore important to investigate if GUVacs are observed in cancer patients, and whether their formation is reversible. Further research is also required to illuminate the molecular mechanisms governing GUVac biology, and to develop molecules that can specifically reverse their creation. Despite these unknowns, it is exciting to consider that fundamentally understanding the cytoskeleton rearrangements that underlie anoikis resistance in cancer may one day translate into therapies that improve human health.
